# Right ventricular myocardial edema leading to severe heart failure after open heart surgery – possible effect of high-dose corticosteroid therapy

**DOI:** 10.1186/s13019-024-02656-4

**Published:** 2024-04-05

**Authors:** Karoline Korsholm Jeppesen, Lars Peter Riber, Jordi Sanchez Dahl

**Affiliations:** 1 Department of Cardiology, Odense University Hospital, Kløvervaenget 47, Odense C, 5000 Denmark; 2 Department of Cardiac, Thoracic and Vascular Surgery, Odense University Hospital, Odense, Denmark

**Keywords:** Aortic dissection, Myocardial edema, Heart failure, Corticosteroid treatment

## Abstract

**Background:**

Cardiopulmonary bypass induces a systemic inflammatory response and alterations in fluid homeostasis, resulting in generalized tissue edema. Additionally, ischemia-reperfusion injury following cardioplegic arrest presumably prompts organ-specific myocardial edema.

**Case presentation:**

The case report presents a 75-year-old Caucasian male diagnosed with aortic dissection, Stanford type A, who underwent complicated open-heart surgery. Postoperatively, the patient developed excessive myocardial edema, particularly affecting the right ventricle myocardium to an extent where the right ventricle surpassed the sternal rim, making it impossible to close the sternum. Ischemia was ruled out by performing coronary angiography, demonstrating well-calibrated coronary arteries. Transoesophageal echocardiography showed a restrictive right ventricle with free-wall thickness of 30 mm, severely reduced right ventricle systolic function and a volume-depleted left ventricle consistent with right ventricular heart failure due to right ventricular edema. The patient presented with unstable haemodynamics despite use of inotropes and continuation of open sternotomy. In an attempt to reduce myocardial edema, the patient was started on corticosteroid treatment despite of ongoing mediastinitis. Corticosteroid treatment reduced myocardial edema and enabled the closure of sternum on the 44th postoperative day.

**Conclusions:**

The case report addresses the clinical relevance of corticosteroid treatment in selective cases of intractable haemodynamically significant postoperative myocardial edema.

## Background

Subsequent to open-heart surgery and haemostasis in the haemodynamically stable patient, the sternal retractor is removed, and the sternum closed. Sternal closure mechanically increases intrathoracic pressure, initiating a cascade of mechanisms. Presumably, the increase in intrathoracic pressure reduces transmural pressure due to an increase in pressure outside the heart, thus decreasing the pressure gradient across the ventricle wall. Hence, compromising left ventricle (LV) diastolic filling and consequently reducing preload and stroke volume [1]. However, a haemodynamically marginal patient is vulnerable to additional impairment. Hence, selective postponement of postoperative sternal closure instigates cardiac recovery and augments cardiac performance in the marginal patient. Postponement is weighed against risk of infection and thoracic instability. Postoperative indications for the continuation of open sternotomy include low cardiac output syndrome, refractory haemorrhage, arrhythmias and cardiac edema [2].

Cardiopulmonary bypass (CPB) induces a systemic inflammatory response and alterations in fluid homeostasis, resulting in generalized tissue edema [3]. In addition, ischemia-reperfusion injury following cardioplegic arrest presumably prompts organ-specific myocardial edema.

This case report addresses the clinical relevance of corticosteroid treatment in selective cases of intractable haemodynamically significant postoperative myocardial edema.

## Case presentation

A 75-year-old Caucasian male, with a history of hypertension, diagnosed with Stanford type A aortic dissection at a peripheral hospital, was transferred to Odense University Hospital for emergency open-heart surgery. The ascending aorta and aortic valve were replaced using a Medtronic Freestyle aortic root prosthesis with reimplantation of coronary arteries, and an aortic prosthesis replaced the distal ascending aorta with reimplantation of truncus brachiocephalicus. In the process of concluding haemostasis at extracorporeal circulation time 258 min and aortic cross-clamp time 148 min, the patient developed circulatory collapse and left ventricle dilatation. Bleeding was identified at the left coronary artery, which was repaired causing the patient to stabilize. Subsequently, the attempted sternal closure provoked recurrent circulatory collapse. Consequently, a vacuum-assisted closure device was established to postpone sternal closure. The patient was admitted in stable condition to the intensive care unit with mean arterial pressure of 55 mmHg on norepinephrine (NE) 0.32 µg/kg/min.

**Fig. 1:**
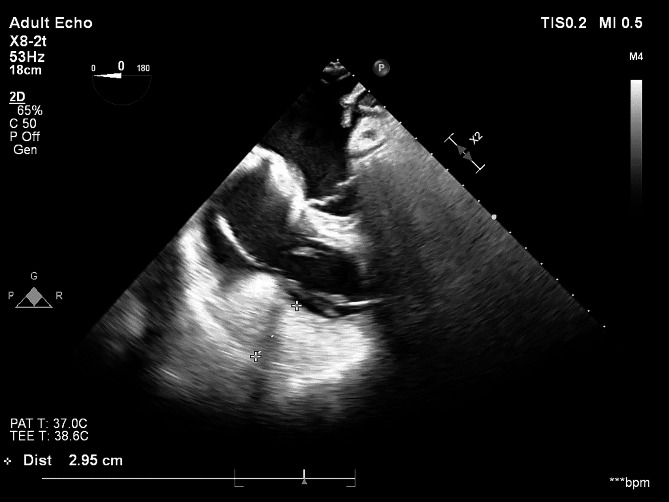
Transoesophageal echocardiography showing right ventricle myocardial thickening with free-wall of 30 mm

The first week postoperative, the patient developed progressive edematous thickening of the myocardium, particularly involving the right ventricle (RV) myocardium to an extent where RV surpassed the sternal rim. Transoesophageal echocardiography (TEE) showed RV free-wall thickness measuring 30 mm (Fig. 1), severely impaired systolic RV function, dilated right atrium with septal deviation towards left atrium, and a seemingly volume-depleted hyperdynamic LV. TEE imaging demonstrated no signs of pericardial effusion and was not compatible with haematoma. Postoperative creatine phosphokinase MB was elevated at 27 µg/l with subsequent rapid decline as expected following surgery. Ischemia was ruled out performing coronary angiography demonstrating well-calibrated coronary arteries. Combined with increased central venous pressure at 20 mmHg, findings were consistent with RV heart failure due to RV myocardial edema.

Haemodynamics were unstable, despite initial treatment with levosimendan (0.1 µg/kg/min) and continuous high-dose NE. The poor hemodynamic status was interpreted as the consequence of severe RV myocardial edema, leading to RV diastolic dysfunction and consequently reduced LV preload. Consequently, treatment with dopamine (3 µg/kg/min) was initiated to augment inotropic and chronotropic cardiac function. Additionally, culture from mediastinum was positive for staphylococcus haemolyticus; therefore, Piperacillin/Tazobactam (4 g/0.5 g 3/day) and Vancomycin (1 g/day for 5 days) were administered. Following a multidisciplinary conference decision, the patient was started on Methylprednisolone in an attempt to reduce the myocardial edema. The patient received Methylprednisolone 1 g/day for 5 days, hereafter 100 mg/day for 5 days, and henceforward the patient was reduced 25 mg every other day. The following week the myocardial edema reduced, and NE requirement decreased substantially (0.01 µg/kg/min).

Nineteen days after initiating corticosteroid therapy, there was no macroscopical sign of infection, and the sternum was closed without cardiac impairment. The following night, the patient developed cardiac tamponade due to haematoma pressing on the right atrium. The sternum was reopened, and the haematoma evacuated. Subsequently, the patient developed renewed backward failure with progressing impairment of liver function. TEE revealed recurrent RV myocardial edema.

Corticosteroid therapy was reinitiated. The patient received Solu-Cortef 125 mg 4/day for 5 days, hereafter 125 mg/day for 4 days, and henceforward slow reduction (50 mg/day for 1 week, 37.5/day for 1 week, 25 mg/day for 1 week, 15 mg/day for 1 week, 10 mg/day for 1 week and 2.5 mg/day for 1 week).

Multiple tests were performed to determine the pathophysiology. Sinus coronarius venogram was normal without obstruction. Right heart catheterization showed biventricular heart failure with an increased pulmonary capillary wedge pressure at 16 mmHg, increased end-diastolic pressure in RV, and reduced cardiac index 2.1 L/min/m^2^. Pulmonary capillary resistance was normal. Myocardial biopsy showed normal myocyte size and was not consistent with myocarditis.

The 44th postoperative day, the sternum was finally closed. Transthoracic echocardiography one month after sternal closure showed insignificant myocardial edema and biventricular systolic function tangent to normal.

## Discussion and conclusions

The possible inhibitory effect of perioperative methylprednisolone on CPB-induced inflammatory response in high-risk patients undergoing cardiac surgery on CPB has been studied in a randomised, double blinded, placebo-controlled trial [4]. In this study, Whitlock et al. [4] reported no statistically significant reduction in mortality or major morbidity in patients receiving perioperative methylprednisolone compared to placebo. Furthermore, no statistically significant difference in the risk of infection was reported [4]. Limited data exists studying the impact of corticosteroid treatment on postoperative-associated myocardial edema.

The systematic review by Dekker et al. [3] investigated the effect of different pharmacological treatments on organ-specific edema following CPB. Myocardial edema, quantified by myocardial wet-to-dry ratio, was reported in 8 of 32 animal studies [3]. Hereof, one study by Farstad et al. [5] investigated the effects of pre-treatment with methylprednisolone (Solu-Medrol 30 mg/kg times 2) in 7 piglets prior to CPB compared to a control group. Farstad et al. [5] did not show a significant reduction of total tissue water content in myocardial tissue in piglets treated with methylprednisolone prior to CPB.

In the present case report, corticosteroid treatment was administered postoperatively and by the indication of intractable myocardial edema. Hence, this case report offers new information by addressing the clinical relevance of corticosteroid treatment in selective cases of intractable haemodynamically significant postoperative myocardial edema.

## Data Availability

Data appear in the written article.

## Abbreviations

LV: Left ventricle
CPB: Cardiopulmonary bypass
NE: Norepinephrine
RV: Right ventricle
TEE: Transoesophageal echocardiography
